# Mismatch repair deficiency and microsatellite instability in adrenocortical carcinoma

**DOI:** 10.1016/j.esmoop.2025.106030

**Published:** 2026-02-05

**Authors:** B. Altieri, S. Kircher, S. Herterich, A. Jahn, M.-V. Teleanu, J. Lippert, L.-S. Landwehr, O. Kimpel, M. Reuter, H. Remde, A. Stenzinger, H. Glimm, R.C. Bargou, S. Fröhling, C.L. Ronchi, S. Appenzeller, M. Fassnacht, M. Kroiss

**Affiliations:** 1 Department of Internal Medicine I, Division of Endocrinology and Diabetes, University Hospital, University of Würzburg, Würzburg, Germany; 2 Bavarian Cancer Research Center (BZKF), University Hospital of Würzburg, Würzburg, Germany; 3 Institute of Pathology, University of Würzburg, Würzburg, Germany; 4 Clinical Chemistry and Laboratory Medicine, University Hospital, University of Würzburg, Würzburg, Germany; 5 Institute for Clinical Genetics, University Hospital Carl Gustav Carus at TUD Dresden University of Technology and Faculty of Medicine of TUD Dresden University of Technology, Dresden, Germany; 6 National Center for Tumour Diseases (NCT), Dresden, Germany; 7 European Reference Networks (ERN) GENTURIS, Hereditary Cancer Syndrome Center Dresden; 8 Division of Translational Medical Oncology, German Cancer Research Center (DKFZ), Heidelberg, Germany; 9 National Center for Tumor Diseases (NCT), NCT Heidelberg, a partnership between DKFZ and Heidelberg University Hospital, Heidelberg, Germany; 10 Institute of Human Genetics, University of Würzburg, Würzburg, Germany; 11 Institute of Pathology, University Hospital of Heidelberg, Heidelberg, Germany; 12 Translational Medical Oncology, University Hospital Carl Gustav Carus, TUD Dresden University of Technology, Dresden, Germany; 13 Comprehensive Cancer Center Mainfranken, University Hospital of Würzburg, Würzburg, Germany; 14 German Cancer Consortium (DKTK), Core Center Heidelberg, Heidelberg, Germany; 15 Institute of Human Genetics, Heidelberg University, Heidelberg, Germany; 16 Department of Metabolism and Systems Science, College of Medicine and Health, University of Birmingham, Birmingham, UK; 17 European Reference Networks (ERN) EURACAN, ERN for all Rare Adult Solid Cancers Expert Centre, Würzburg, Germany; 18 Department of Internal Medicine IV, University Hospital Munich, Ludwig-Maximilians-Universität München, Munich, Germany

**Keywords:** MMR deficiency, MSI, immunohistochemistry, methylation, immune checkpoint inhibitors, Lynch syndrome

## Abstract

**Background:**

Genetic and epigenetic alterations can cause mismatch repair (MMR) deficiency (dMMR) leading to microsatellite instability (MSI). Although dMMR/MSI predicts response to immune checkpoint inhibitors (ICIs) in several cancers, their relevance in adrenocortical carcinoma (ACC) remains unclear.

**Patients and methods:**

We investigated the MMR system and MSI in patients with apparently sporadic ACC and explored associations with clinical characteristics and outcomes. In a subgroup, correlation between dMMR/MSI and response to ICI was evaluated.

Immunohistochemistry for MLH1, PMS2, MSH2, and MSH6 was carried out in 109 ACC tissues with molecular data. Germline pathogenic/likely pathogenic (P/LP) and somatic oncogenic/likely oncogenic (O/LO) MMR variants were validated by Sanger sequencing. *MLH1* methylation and *EPCAM* deletions were assessed via multiplex ligation-dependent probe amplification. MSI was analysed using plex PCR.

**Results:**

dMMR was identified in 15 (14%) cases, mainly involving MSH6 loss (*n* = 9, 8.3%) either with MSH2 or alone. No significant differences were observed in hormone secretion, European Network for the Study of Adrenal Tumors (ENSAT) stage, proliferation index (Ki67%), S-GRAS (Stage, Grade, Resection status, Age, Symptoms) score, progression-free survival (8 versus 14 months) and overall survival (72 versus 80 months) between patients with and without dMMR. A slightly higher frequency of other malignancies was observed in dMMR cases (27% versus 11%, *P* = 0.09). Ten dMMR tumours were linked to P/LP germline (*n* = 4, 26.7%) or O/LO somatic (*n* = 5, 33.6%) MMR variants or *MLH1* hypermethylation (*n* = 1, 6.7%), but only three (20%) showed MSI. Lynch syndrome was identified in 5% of patients. Among 12 patients treated with ICIs, time to progression was similar between those with and without defective MMR (4 versus 5 months, *P* = 0.21). Only one of five ICI responders had a confirmed *MSH6* variant.

**Conclusion:**

DMMR occurs in a minority of ACC, often without MSI. Although Lynch syndrome accounts for a subset of cases, dMMR is not predictive of clinical features or ICI response. Nonetheless, MMR testing remains important for identifying individuals at hereditary cancer risk.

## Highlights

• dMMR was found in 14% of ACC, mainly showing loss of MSH6 and MSH2 protein expression.
• In one-third of dMMR ACC cases, a causative genetic or epigenetic alteration was not identified.
• MSI is infrequent in ACC even in patients with dMMR at immunohistochemistry.
• dMMR was not associated with clinicopathological characteristics, prognosis, and response to immunotherapy.
• Immunohistochemistry remains the preferred first-line screening tool for Lynch syndrome in ACC.

## Introduction

Advanced adrenocortical carcinoma (ACC) counts among the most difficult treatable endocrine malignancies with a response rate to standard therapy with mitotane and platinum-based therapy of only 20%-25% and median survival of only 11-15 months.^1^, ^2^, ^3^

Given the limited efficacy of second-line treatments,^4^ immunotherapy has emerged as a potential alternative.^5^ Clinical trial results for immune checkpoint inhibitors (ICIs) in ACC have been heterogeneous, with median progression-free survival ranging from 2 to 7 months.^6^, ^7^, ^8^, ^9^, ^10^ A real-world response rate of 14.5% has been reported,^11^ consistent with findings from a recent meta-analysis.^12^ The combination of ICI with other therapies may increase response rates.^13^^,^^14^ The combination of the programmed cell death protein 1 [PD-(L)1] inhibitor camrelizumab with the vascular endothelial growth factor receptor inhibitor apatinib was reported to lead to an unprecedented objective response rate of 52%.^13^ Although some patients may derive clinical benefit from ICI,^15^ markers of response are lacking. PD-L1 expression, a biomarker of ICI response in other tumours,^16^ is expressed at low levels in ∼25% of ACC.^17^ However, it did not predict response in prospective clinical trials,^6^^,^^8^^,^^9^ and had limited predictive value in a real-world series.^11^ Similarly, tumour mutational burden (TMB) was shown to be irrelevant as a response marker in ACC.^18^

DNA mismatch repair (MMR) is essential for genomic integrity and stability.^19^^,^^20^ Inactivation of MMR genes may occur due to germline or somatic variants or epigenetic silencing. This inactivation results in loss of expression of MMR proteins, termed MMR deficiency (dMMR). dMMR leads to impaired DNA repair, which results in increased TMB and microsatellite instability (MSI). Tumours with dMMR/MSI have been reported to respond more frequently to ICIs than tumours with intact MMR (MMR proficient, pMMR).^21^ This prompted tumour-agnostic approval of pembrolizumab for dMMR/MSI tumours.^22^^,^^23^

Lynch syndrome (LS) is an autosomal dominant hereditary cancer syndrome caused by heterozygous pathogenic germline variants in MMR genes. LS is associated with high lifetime risk for colorectal, endometrial, and ovarian cancers, and a lower risk for other tumours including ACC.^24^^,^^25^ Evidence linking LS to ACC is limited. In the study by Raymond et al., of 94 ACC patients, 3 had LS, and among 135 LS patients, 2 had ACC; most tumours had microsatellite stability (MSS) despite MMR protein loss.^26^ Another study of 634 patients with LS identified only 3 cases (0.47%) of ACC, all with MSH2 and MSH6 loss.^27^ More robust data are needed to determine the role of MMR and LS screening in ACC and the impact MMR and LS may have on ICI therapy.

The aim of this study was to comprehensively evaluate MMR protein expression, MSI status, germline and somatic MMR variants and methylation, and *EPCAM* deletion in a large ACC cohort. We also intended to correlate findings with clinicopathological characteristics, including clinical outcomes and response to immunotherapy.

The study is reported in accordance with the Reporting Recommendations for Tumor Marker Prognostic Studies (REMARK) reporting checklist (Supplementary Table S1, available at https://doi.org/10.1016/j.esmoop.2025.106030).^28^

## Patients and methods

### Patients

**Figure 1:**
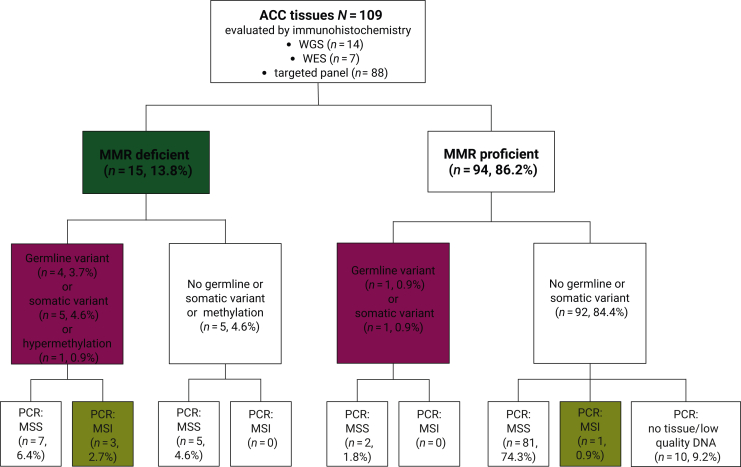
**Flow diagram visualising the results of immunohistochemistry of MMR proteins, molecular genetics, and microsatellite analysis.** Immunohistochemistry (IHC) was carried out in all 109 tissue samples, showing loss of MMR protein expression (MMR deficiency, dMMR) in 13.8% of cases. Germline pathogenic/likely pathogenic (P/LP) variants, indicative of Lynch syndrome, were present in four patients with dMMR and one patient without. Four patients with MSI were detected in this cohort, three in the group with dMMR (left branch) and one in the group with MMR proficiency (pMMR, right branch). MMR, DNA mismatch repair; MSI, microsatellite instability; MSS, microsatellite stability; WES, whole exome sequencing; WGS, whole genome sequencing.

The study participants and study assessments are shown in Figure 1. One hundred and nine patients with histologically proven ACC and available data on MMR variants by whole genome (*n* = 14), exome (*n* = 7) or targeted panel sequencing (*n* = 88) and available formalin-fixed paraffin-embedded (FFPE) tumour material were included. Tumour material derived from surgery of primary tumour (*n* = 83), or surgery or biopsy of local recurrence (*n* = 9) or distant metastasis (*n* = 17). The study protocol was conducted in accordance with the Declaration of Helsinki. All patients were part of the European Network for the Study of Adrenal Tumors (ENSAT) registry study approved by the ethics committee of the University of Würzburg (#88/11) and provided written informed consent. The ENSAT registry is a longitudinal, observational registry that systematically collects clinical, pathological, and treatment data, and clinical outcomes and biological specimens from patients with adrenal tumours.^29^ It provides a comprehensive resource to advance understanding of adrenal tumourigenesis and to inform strategies for optimizing patient management.

Fourteen patients were additionally included in the DZKF/NCT/DKTK MASTER program (Molecular Stratification for Tumor Eradication Research), a multicentre, prospective study that is based on a common workflow for diagnostics, therapeutic decision making, and structured follow-up in patients with rare tumours failing standard treatment^30^^,^^31^ approved by the local ethics committee and by the ethics committee of the University of Heidelberg (S-206/2011).

Clinical and histopathological characteristics at the time of first diagnosis including age, sex, tumour size, hormone excess, ENSAT tumour stage,^32^ tumour resection status, Weiss score, Ki67 proliferative index, S-GRAS (Stage, Grade, Resection status, Age, Symptoms) score,^33^ and history of other tumours and clinical outcome (including response to ICI treatment) were retrospectively collected from medical records. Last follow-up was done in December 2024. Family history of additional tumour types was collected to assess fulfilment of the Amsterdam II criteria for LS diagnosis.^34^

### Immunohistochemistry

Immunohistochemistry (IHC) for MLH1 (clone G168-15, BD Transduction Laboratories, 1 : 200), MSH2 (clone Fe11, Calbiochem, 1 : 40), MSH6 (clone EP49, Epitomics, 1 : 1000), and PMS2 (clone A16-4, BD Biosciences, 1 : 400) was carried out on 109 FFPE tissue slides of ACC according to standard protocols. All immunoperoxidase-labelled sections were counterstained for 2 min with haematoxylin (Sigma-Aldrich, Taufkirchen, Germany). The expression of MMR proteins MLH1, MSH2, MSH6, and PMS2 were assessed as described before.^35^ Nuclear staining of surrounding stromal and immune cells was applied as internal positive control.

Classical loss of heterodimers MLH1-PMS2 or MSH2-MSH6 as well as unclear isolated loss of PMS2 or MSH6 by IHC were considered to be dMMR.^36^, ^37^, ^38^

### Sample collection, DNA isolation and sequencing

For whole genome sequencing (WGS) and targeted panel next-generation sequencing (NGS), DNA was isolated from fresh-frozen (*n* = 14) or FFPE (*n* = 88) tumour specimens and matched blood samples as previously described.^30^^,^^31^^,^^39^ For whole exome sequencing (WES), DNA from seven fresh-frozen tumour and matched blood samples was extracted using the Maxwell® RSC DNA Kit (#AS1400, Promega, Walldorf, Germany), according to manufacturer’s instructions.

Quality control and quantification of DNA samples was assessed using GeneRead DNA QuantiMIZE_384_DataAnalysis (Qiagen, Hilden, Germany) or Qubit Fluorometer (Fischer Scientific, Schwerte, Germany), a 4200 or 2200 TapeStation system (Agilent Technologies, Waldbronn, Germany), and a 2100 Bioanalyzer system (Agilent Technologies), following manufacturer’s instructions.

Details on DNA sequencing, including WGS, targeted NGS, and WES and bioinformatic analysis are reported in the Supplementary Appendix, available at https://doi.org/10.1016/j.esmoop.2025.106030. Independently from the method of the sequencing, all variants were evaluated according to the ACMG/ClinGen and VICC criteria.^40^^,^^41^ Only pathogenic/likely pathogenic (P/LP) germline variants and oncogenic/likely oncogenic (O/LO) somatic variants in MMR genes were further analysed. P/LP and O/LO variants in MMR genes, as well as variants of uncertain significance (VUS) in *MSH2* and *MSH6* in patients with dMMR, were additionally validated by Sanger sequencing. Details on the method and the used primers are reported in Supplementary Appendix and Table S2, available at https://doi.org/10.1016/j.esmoop.2025.106030. Intronic copy number variation resulting in intronic duplication of the *MSH6* gene was validated with multiplex ligation-dependent probe amplification (MLPA) method (see next paragraph).

### MMR methylation status and deletion

In patients with dMMR but without evidence of P/LP or O/LO variants of MMR genes, methylation status and deletions in promoter regions of *MLH1*, *MSH2*, *PMS2*, and *MSH6* and deletion in the 3′ region of *EPCAM* was assessed by MLPA using the SALSA MLPA probe mix ME011 and P072-D1 (MRC Holland, Amsterdam, The Netherlands) according to the manufacturer’s protocol. For each MS-MLPA reaction 150 ng of genomic DNA were used. Hybridisation, ligation and PCR amplification of probes were carried out in a Biometra 96-well PCR thermocycler. Fragment separation by capillary electrophoresis was carried out on a CEQ8000 capillary sequencer (ABSciex, Framingham, MA). The obtained peaks were size-called and assigned to MLPA-probes by GenomLab GeXP-Fragment Analysis software (software version 10.2.3). MLPA quality control fragments included DNA concentration, MLPA-reaction, DNA-denaturation, and HhaI digestion. Quantification was carried out using the peak height in relative fluorescence units. After normalisation, dosage quotients of each peak were evaluated and compared with amplification products of digested and undigested samples.

### Microsatellite instability

MSI analysis was carried out in 99 out of 109 tissues with available tumour DNA and compared with matched DNA from peripheral white blood cells. MSI analysis was conducted using mononucleotide and dinucleotide microsatellite targets *NR-21*, *NR-27*, *BAT40*, and *KCNJ5* in a plex PCR system. Primer sequences are given in Supplementary Table S3, available at https://doi.org/10.1016/j.esmoop.2025.106030. PCR was carried out in a Biometra 96-well PCR thermal cycler (30 cycles at 94°C for 30 s, 60°C for 30 s and 72°C for 30 s, with a final extension at 72°C for 10 min). Fragment separation was done by capillary electrophoresis on a CEQ8000 capillary sequencer (ABSciex, Framingham, MA). Peaks are size-called and assigned to marker-alleles by GenomLab GeXP-Fragment Analysis software (software version 10.2.3).

*NR-21*, *NR-27*, and *BAT40* are quasi-monomorphic and obviate the need for matched healthy tissue derived DNA. MSI was detected by comparing tumour-derived allele variation range of each marker with allele variation range in any MSS DNA. MSI of the dinucleotide marker *KCNJ5* was defined by additional stutter fragments of the observed alleles. MSI was defined as loss of stability in at least one of the four evaluated microsatellite markers and considered MSI-low or -high if the instability was found in only one locus or at least two loci, respectively. MSS was defined by the absence of instability in the investigated loci.

### Statistical analysis

Continuous variables were evaluated by the Shapiro–Wilk test and reported as median with lower and upper quartile (Q1-Q3). Categorical variables are displayed as numbers and percentages. Patients with dMMR were compared with those who were pMMR. A two-sided Student's *t*-test or Mann–Whitney test was used to compare continuous variables and Fisher’s exact test or chi-square test was used for categorical variables. Progression-free survival (PFS) was calculated as the time (in months) from primary diagnosis to the first documented disease progression or the date of last follow-up. Overall survival (OS) was calculated as the time (in months) from primary diagnosis to patient death or last follow-up. Both PFS and OS were evaluated separately in patients with local diseases (ENSAT tumour stage I-III) and those with advanced disease (ENSAT tumour stage IV) at primary diagnosis. Kaplan–Meier plots with log-rank tests were computed to assess survival analysis. Univariate and multivariate Cox regression analysis was carried out to calculate hazard ratios (HR) and 95% confidence intervals (CI) for PFS and OS. Factors associated with *P* < 0.10 at univariate analysis were further evaluated at multivariate regression. MMR status was included in the multivariate analysis because it represents the objective of this study. Time to progression (TTP) under therapy was defined as the time (in months) from the start of the ICI until first evidence of disease progression according to RECIST v.1.1,^42^ which was also used to define objective response.

The analysis of the data was carried out using SPSS v.29 (IBM SPSS Statistics) and GraphPad Prism v.9.0 (La Jolla, CA). A *P* value < 0.05 was considered statistically significant.

## Results

### dMMR and MSI in an unselected ACC cohort

The results of the combined analyses are summarised in Figure 1. Among the 109 ACC tissue samples studied, 15 (13.8%) showed evidence of dMMR detected by IHC.

**Figure 2:**
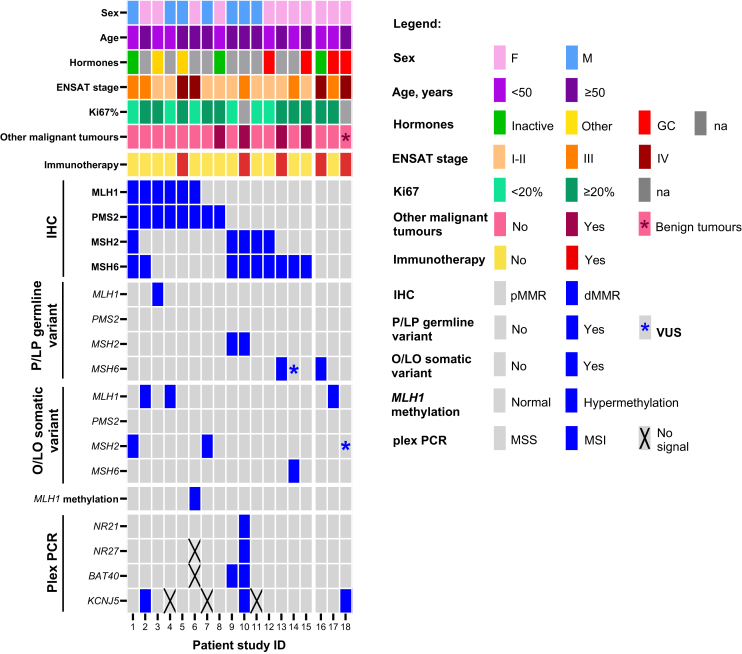
**Global visualisation of clinical and molecular results in patients with a defective MMR mechanism.** Clinicopathological data including patient history of other tumours are given as colour-code. dMMR, deficient MMR protein expression; ENSAT, European Network for the Study of Adrenal Tumors; GC, glucocorticoids; IHC, immunohistochemistry; MSI, microsatellite instability; MSS, microsatellite stability; na, not available; O/LO, oncogenic/likely oncogenic variant; P/LP, pathogenic/likely pathogenic variant; pMMR, proficient MMR protein expression; VUS, variant of uncertain significance.

Figure 2 provides an overview of the molecular characteristics in patients with dMMR (*n* = 15) and in the three patients without loss of any MMR protein by IHC (pMMR) but who had germline P/LP (*n* = 1) or somatic O/LO (*n* = 1) variant in the MMR gene or MSI by plex PCR (*n* = 1), together with clinical and pathological data.

**Figure 3:**
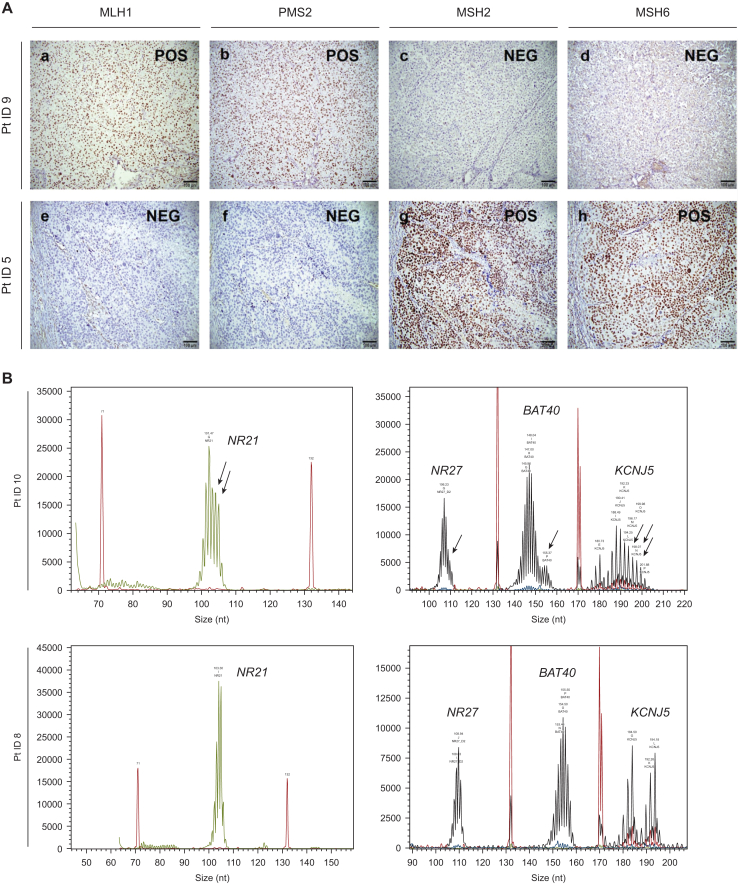
**(A) Immunohistochemistry for MMR protein and (B) analysis of microsatellite instability by plex PCR.** (A) Paradigmatic cases where expression of MMR proteins is lost in a pair-wise manner as heterodimer MSH2-MSH6 protein loss and retained expression of MLH1 and PMS2 [top panel, patient (pt) ID #9, a-d] and as heterodimer MLH1-PMS2 loss with retained MSH2 and MSH6 expression (lower panel, patient ID #5, e-h). Scale bars: 100 μm. (B) Single case with microsatellite instability (MSI)-high in all four investigated loci (patient ID #10) in comparison with a paradigmatic case with microsatellite stability (MSS). Arrows insdicate micrisatellite instabilitiy loci. NEG, negative; POS, positive.

A total of 10 out 15 cases (9.2% of total sample) presented the classic protein loss of heterodimers MLH1-PMS2 or MSH2-MSH6, whereas 5 cases (4.6%) had isolated loss of MSH6 or PMS2 protein. Loss of MSH6 protein expression was predominant (9 cases, 8.3%, Figure 2) and occurred as heterodimer together with loss of MSH2 expression (*n* = 4, 3.7%, Figure 3A a-d), or as isolated loss (*n* = 3, 2.8%), or together with both MLH1 and PMS2 loss (*n* = 1, 0.9%). In one case, loss of expression affected all MMR-proteins (0.9%). MSH1-PMS2 heterodimer loss occurred in six cases (5.5%, Figure 3A e-h), whereas isolated loss of was found in an additional two cases (Figure 2).

Germline P/LP variants, variants of uncertain significance (VUS), and somatic O/LO variants of MMR genes are summarised in Supplementary Table S4, available at https://doi.org/10.1016/j.esmoop.2025.106030. All the reported variants were confirmed by Sanger sequencing or by MLPA. LS, caused by germline P/LP variants in MMR genes, was found in five cases (4.6% of the population). Three (2.8%) of them had a family history that met the clinical Amsterdam II criteria for the diagnosis of LS, whereas family history was unknown in the remaining two subjects (1.8%). Somatic O/LO variants in MMR genes were reported in six (5.5%) cases. Considering the entire cohort, MSI was found in a total of four (4%) ACC, including one case of MSI-high and three cases of MSI-low.

Considering the subgroup of patients with dMMR detected by IHC, 10 of 15 patients had P/LP germline (*n* = 4, 26.7%) or O/LO somatic (*n* = 5, 33.3%) MMR variants or *MLH1* promoter hypermethylation (*n* = 1, 6.7%), but plex PCR showed MSI only in 3 (20%) of these cases (Figures 1 and 2). Five cases (33.3%) with dMMR showed no P/LP germline or O/LO somatic variant, no hypermethylation nor deletions in the promoter regions of MMR genes and no deletion of *EPCAM*. P/LP germline variants were most frequently found in *MSH2* and *MSH6* genes (Figure 2 and Supplementary Table S4, available at https://doi.org/10.1016/j.esmoop.2025.106030). We found O/LO somatic variants in *MLH1* and *MSH2* in two cases, respectively, and in *MSH6* in one case (Figure 2 and Supplementary Table S4, available at https://doi.org/10.1016/j.esmoop.2025.106030). No P/LP germline or O/LO somatic variants were found in *PMS2*. Loss of the heterodimer MSH2-MSH6 protein expression was associated with P/LP variant in the MSH2 gene in two of four cases. Among these cases was the single patient (ID #10) who presented MSI in all four investigated microsatellite targets (MSI-high, Figure 3B) associated with the germline pathogenic variant in *MSH2* (c.1552_1553delCA). Of note, this patient was the only one within the investigated cohort with a previous history of colon cancer. On the other hand, the single patient who showed a tetra-loss of MMR protein expression (ID #1) had a somatic oncogenic variant in *MSH2* (c.1408del) (Figure 2) but was MSS at PCR. Loss of the heterodimer MLH1-PMS2 protein expression was associated with the germline P/LP variant in *MLH1* (c.790+4A>G) in one case, with somatic O/LO variants (c.2179_2182del and c.1420del) in two of three cases, and *MLH1* promoter hypermethylation; in one case, this was additionally linked to MSH6 loss. Isolated protein loss of MSH6 was associated with germline or somatic variants in the MSH6 gene in two of three cases (Figure 2). Of note, one of the two cases with isolated PMS2 loss (ID #7) had an oncogenic somatic variant in *MSH2* (c.2251G>A) together with a likely benign germline variant in *PMS2* (NM_000535.7:c.1688_1689delinsAG, p.Arg563Gln).

In the pMMR group (*n* = 94), we found one patient with a pathogenic germline variant in *MSH6* (c.1238G>A), one who had a somatic (likely oncogenic) variant in *MLH1* (c.G1750C), and one patient who presented with MSI-low associated with two different VUS in *MSH2* (c.1748A>G and c.2224G>A) (Figure 3 and Supplementary Table S4, available at https://doi.org/10.1016/j.esmoop.2025.106030). Of note, the patient with the germline *MSH6* variant was one of the three patients who fulfilled the LS clinical criteria.

No differences in the loss of MMR protein or the presence of somatic O/LO variants between primary and recurring disease or metastasis were found (*P* = 0.11 and *P* = 0.57, respectively).

### Association of dMMR with clinical parameters

To determine whether dMMR correlated with clinical parameters, we compared the group of 15 patients with tumoural loss of at least one MMR protein with all other patients (Table 1). No significant differences were observed in clinical and pathologic parameters, except for the Weiss score, which was higher in the dMMR group (*P* = 0.008) (Table 1). Patients with dMMR had a trend toward a higher prevalence of history of other malignancy compared to those with pMMR (26.7% versus 10.9%, *P* = 0.09). The small number of patients with germline variants (5/109) precluded a separate comparison of this group.

**Table 1:** Clinical parameters at diagnosis in patients with or without evidence of dMMR detected by immunohistochemistry, mutational analysis and PCR

Parameters	dMMR	pMMR	*P* value, χ^2^
Total number of patients	15	94	
Sex	15	94	
Female	8 (53.3)	56 (59.6)	0.65, 0.21
Male	7 (46.7)	38 (40.4)	
Age at diagnosis, years	51 (44-58)	48 (38-57)	0.45
Tumour size, cm	13.5 (8-17)	11 (8-14.8)	0.19
Hormone excess	6	72	
Glucocorticoids ± others	2 (33.3)	45 (62.5)	0.31, 2.36
Other steroids	2 (33.3)	10 (13.9)	
Inactive	2 (33.3)	17 (23.6)	
ENSAT tumour stage at diagnosis	15	94	
I-II	9 (60.0)	45 (47.9)	0.55, 1.19
III	4 (26.7)	25 (26.6)	
IV	2 (13.3)	24 (25.5)	
Resection status	14	92	
R0-RX	12 (85.7)	63 (68.5)	0.38, 1.91
R1-R2	2 (14.3)	25 (27.2)	
No surgery	0	4 (4.3)	
Ki67%	14	87	
Median value	25 (9-55)	15 (8-20)	0.12
Weiss score	13	77	
Median value	7 (6-8)	6 (5-7)	**0.008**
S-GRAS score	13	88	
0-1	1 (7.7)	10 (12.2)	0.47, 2.54
2-3	8 (61.5)	33 (40.2)	
4-5	1 (7.7)	18 (22.0)	
6-9	3 (23.1)	21 (25.6)	
History of other malignant tumours	15	92	
Yes	4 (26.7)	10 (10.9)	0.09, 2.83
No	11 (73.3)	82 (89.1)	

Number of cases with data available is shown for each parameter. Unknown data were excluded from the analyses. Continuous data are reported as median (Q1-Q3), whereas categorical variables are reported as number (percentage).

dMMR, MMR deficient; MMR, DNA mismatch repair; pMMR, MMR proficient; S-GRAS, Stage, Grade, Resection status, Age, Symptoms.

**Figure 4:**
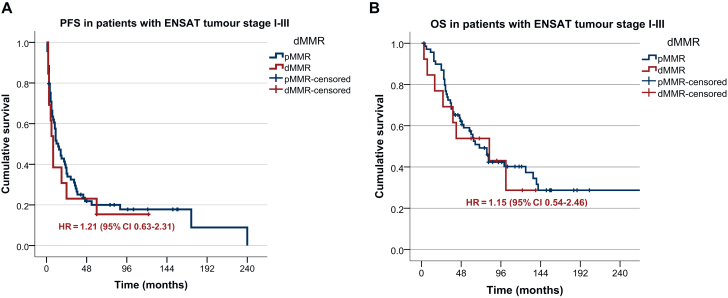
**(A) Progression-free survival (PFS) and (B) overall survival (OS) in patients with and without evidence of MMR deficiency.** Based on immunohistochemistry results, patients were classified as those with DNA mismatch repair (MMR) deficiency (dMMR) or proficiency (pMMR). CI, confidence interval; ENSAT, European Network for the Study of Adrenal Tumors; HR, hazard ratio.

We then compared the prognosis of patients with and without evidence of dMMR at IHC. In patients with ENSAT tumour stage I-III, median PFS was 14 months (95% CI 7.74-20.26 months) in the pMMR group and 8 months (95% CI 4.56-11.44 months) in patients with evidence of dMMR (log-rank *P* = 0.55) (Figure 4A). Median OS was 70 months (95% CI 51.15-88.84 months) in patients with pMMR and 82 months (95% CI 13.48-150.52 months) in those with dMMR (log-rank *P* = 0.71) (Figure 4B). None of the patients died of another malignancy.

Cox regression analysis confirmed the absence of a significant impact of dMMR on clinical outcomes, considering both PFS and OS (Supplementary Tables S5 and S6, available at https://doi.org/10.1016/j.esmoop.2025.106030, respectively). The very small number of patients with dMMR and ENSAT tumour stage IV (*n* = 2) precluded an evaluation of the impact of MMR protein expression on clinical outcomes in advanced stage.

We also evaluated the impact on prognosis of LS. In patients with ENSAT tumour stage I-III, median PFS in patients with LS (*n* = 4) was 18 months (95% CI 2.32-33.68 months) compared with 11 months (95% CI 5.29-16.70 months) in those without (log-rank *P* = 0.98) (Supplementary Figure S1A, available at https://doi.org/10.1016/j.esmoop.2025.106030). Median OS was 82 months (95% CI 23.20-140.80 months) in patients with LS and 70 months (95% CI 50.95-89.04 months) in those without (log-rank *P* = 0.86) (Supplementary Figure S1B, available at https://doi.org/10.1016/j.esmoop.2025.106030). Because of the small number of the patients with LS, we did not perform multivariate regression analysis.

### Response to immunotherapy

From the entire cohort, 12 patients (11%) with advanced disease received ICI: pembrolizumab monotherapy (*n* = 8, 7.3%) or in combination with lenvatinib (*n* = 1, 0.9%), nivolumab monotherapy (*n* = 1, 0.9%) or in combination with ipilimumab (*n* = 1, 0.9%), and avelumab monotherapy (*n* = 1, 0.9%).

Considering patients treated with ICIs, three had dMMR (patient IDs #5, #10, and #13), one had a P/LP variant in *MSH6* but with pMMR (patient ID #16), and one had MSI but without dMMR or P/LP variants in MMR genes (patient ID #18) (Figure 2). Four out of five of these patients (80%) did not respond at all. A single patient (20%) with a germline large duplication in *MSH6* (NC_000002.11:48029043_48032607dup), MSH6 protein loss, but without MSI (patient ID #13), was stable on nivolumab + ipilimumab and had progression after 12 months. In the group without a defective MMR mechanism due to germline and/or somatic variance or epigenetic silencing or MMR protein loss (*n* = 7), there were two patients (20.6%) with partial response and two (20.6%) with stable disease.

Median TTP under ICI treatment was similar between patients with a pMMR mechanism (5 months, 95% CI 2.43-7.57 months) and those with dMMR, MMR gene variants, or MSI (4 months, 95% CI 3.12-4.88 months, log-rank *P* = 0.21; Supplementary Figure S2, available at https://doi.org/10.1016/j.esmoop.2025.106030).

## Discussion

This is the first large systematic study providing a comprehensive perspective of the MMR system in patients with ACC with and without pathogenic/likely pathogenic variants in MMR genes using multiple methods. Considering the entire cohort of ACC, we found that the proportion of dMMR detected by IHC is 14%, with a high prevalence of MSH6 loss (60% of dMMR cases) partly associated with MSH2 loss (27% of dMMR cases). The presence of dMMR was not associated with any of the investigated clinical and pathological characteristics, including tumour-related hormone secretion, tumour aggressiveness (Ki67 proliferation index, ENSAT tumour stage), and clinical outcomes. Of the 15 dMMR cases, 10 (67%) were linked to germline P/LP or somatic O/LO MMR gene variants, or *MLH1* promoter hypermethylation in one case. The remaining 33% had no identifiable genetic or epigenetic cause. Abnormal methylation status of the other MMR genes or deletion of *EPCAM* have not been found in any of the evaluated ACC with dMMR.

In the combined dataset of 203 patients with ACC (comprising our cohort and the one reported by Raymond et al.^26^), the prevalence of LS is 3.9%, supporting previous evidence that a subset of apparently sporadic adult ACC cases might be associated with LS. A summary of all studies and case reports is provided in Supplementary Table S7, available at https://doi.org/10.1016/j.esmoop.2025.106030.^26^^,^^27^^,^^43^, ^44^, ^45^, ^46^, ^47^, ^48^, ^49^, ^50^, ^51^, ^52^, ^53^, ^54^, ^55^ Conversely, the prevalence of ACC in LS ranges from 0.5%-1.5%^26^^,^^27^ (Supplementary Table S7, available at https://doi.org/10.1016/j.esmoop.2025.106030), which is comparable to the incidence of other less common tumours within the LS spectrum.^24^^,^^25^ These observations underscore the importance of identifying patients with LS, as early diagnosis can enable timely tumour detection in both patients and their relatives, ultimately improving clinical outcomes.^56^

In our cohort, the most frequent P/LP germline variants were in *MSH2* and *MSH6*, consistent with previous studies^26^^,^^27^^,^^43^, ^44^, ^45^, ^46^, ^47^, ^48^, ^49^, ^50^, ^51^, ^52^, ^53^, ^54^, ^55^ (Supplementary Table S7, available at https://doi.org/10.1016/j.esmoop.2025.106030). No P/LP germline variants in *PMS2* have been identified in ACC to date. P/LP germline *MMR* variants often correlate with protein loss detected by IHC. Across all reported cases (Supplementary Table S7, available at https://doi.org/10.1016/j.esmoop.2025.106030), P/LP *MSH2* variants were associated with *MSH2* loss (alone or with *MSH6*) in 93% of cases, *MSH6* variants with *MSH6* loss in ∼50%, and *MLH1* variants with *MLH1-PMS2* loss in all cases. Although we also found a case of LS associated with proficient MMR protein expression, as previous reported cases,^26^^,^^55^ IHC could be used as a screening tool to identify patients at risk of LS. Discrepancies between genomic profiling and IHC in some cases may reflect P/LP variants that alter protein function without affecting its expression.

Beyond LS, we identified additional causes of dMMR, including the first ACC case with *MLH1* promoter hypermethylation, associated with MLH1-PMS2 loss, and somatic O/LO variants in MMR genes in five others, accounting for 40% of dMMR ACC cases in our cohort. Distinguishing LS from cases due to *MLH1* hypermethylation or somatic MMR variants is clinically relevant for both cancer risk management and family screening.^57^ Recent evidence shows that *MLH1* promoter hypermethylation and germline MMR variants can co-occur,^58^ suggesting LS should not be excluded solely based on hypermethylation. Tumours with dMMR or MSI but without germline MMR variants were referred to as ‛Lynch-like syndrome’.^57^ In our cohort, somatic O/LO MMR variants were found in 5.5% of cases, most commonly in *MLH1* (50%). This contrasts with Pozdeyev et al., who reported a slightly higher prevalence (8.8%), mainly involving *MSH2* and *MSH6*.^59^ Notably, in our series, one tumour with a somatic *MSH2* variant showed loss of all four MMR proteins, and one with an *MLH1* variant showed loss of *MLH1*, *PMS2*, and *MSH6*.

In a substantial proportion of dMMR cases (*n* = 5), no identifiable genetic or epigenetic cause could be found. Possible explanations include atypical or cryptic pathogenic variants in MMR genes undetectable by current methods or currently classified as VUS, mosaicism of *de novo* variants, or germline variants in non-MMR genes.^60^, ^61^, ^62^ Moreover, technical artefacts found at IHC, especially in those cases (two out five) presenting a single protein loss, cannot be entirely ruled out since these cases were not rechecked.^63^^,^^64^ However, dMMR without detectable germline, somatic, or epigenetic alterations has also been reported in other cancer types.^65^

LS is typically associated with MSI in tumours. In our cohort, however, MSI was detected by PCR in only 4% of ACC cases, with only two being associated with germline MMR variants, including one MSI-high case associated with an *MSH2* variant and one associated with *MSH2* together with *MSH6* germline variants. To note, MSI-low was also found in one patient with *MLH1* somatic variant and one with a VUS somatic variant in *PMS2*. These findings align with previous reports, which also describe rare MSI detection in ACC, including cases with *MSH2* P/LP germline variants^27^ as well as cases where somatic variants in MMR genes have been investigated^59^ (Supplementary Table S7, available at https://doi.org/10.1016/j.esmoop.2025.106030). Although a misclassification of MSS cases by PCR especially in those tumours with isolated MSH6 or PMS2 loss cannot be excluded,^66^, ^67^, ^68^ the prevalence of MSI in our cohort matches that reported by Bonneville et al. (4.3%) using exome sequencing data based MSI calling (MANTIS) from TCGA data,^69^ and is lower than rates in other LS-associated cancers.^69^, ^70^, ^71^ Raymond et al. suggested that events in MMR genes may occur in the late stage of ACC carcinogenesis, preventing the accumulation of detectable MSI.^26^ It is a well-accepted concept that MSI is associated with high TMB, increased neoantigen presentation, and activation of the cyclic GMP-AMP synthase-stimulator of interferon genes signalling,^72^ which promotes immune recognition.^73^ Bonneville et al. observed a higher somatic mutation load in MSI versus MSS ACC.^69^ However, because of the different methods of sequencing, we were not able to use a consistent TMB threshold across samples within our cohort. In addition to that, MSI has been associated with clinical outcomes in different cancers. Particularly, in colorectal cancer, MSI is linked to better prognosis in early-stage disease (∼15% MSI prevalence),^74^ though this benefit may not extend to advanced stages.^74^^,^^75^ It must be acknowledged that the subgroup of MSI cases in our study was too small to be statistically analysed, although disease stage and clinical outcome did not differ significantly between dMMR and pMMR cases.

Interestingly, no clear association between dMMR and response to ICIs was observed in the small subset of investigated patients, consistent with prior clinical trials and our earlier case series,^11^ whereas Raj et al. reported dMMR in two of nine pembrolizumab responders.^9^ ACC does not appear as a primary candidate for ICI treatment. Factors such as tumoural glucocorticoid excess may contribute to an overall immune cell depleted microenvironment of ACC.^17^^,^^76^ Nonetheless, ∼15% of ACC patients respond to ICIs, even without dMMR/MSI, supporting its role as a potential second-line therapy where available.^5^

Although our study is the largest series investigating the MMR system and microsatellite status in patients with apparently sporadic ACC, it has some limitations, foremost that of its retrospective design. Moreover, although this study was conducted at a single tertiary referral centre, the cohort is comparatively large given the extreme rarity of ACC (0.7-2 cases per million per year), and our findings are consistent with previous reports.^26^^,^^27^^,^^43^^,^^59^ Therefore, the results are likely generalisable to a broader context. Only samples with available genomic data and FFPE material were tested for further analysis, introducing potential bias. Additionally, the use of multiple sequencing platforms (targeted NGS, WGS, WES) precluded consistent TMB assessment. However, all P/LP germline and O/LO somatic variants were validated by Sanger sequencing. Notably, unlike prior studies, our analysis included both dMMR-positive and -negative cases.

Our findings highlight the complexity of MMR assessment in ACC, with a substantial proportion of dMMR cases lacking identifiable genetic or epigenetic causes. IHC remains the most practical and effective first-line screening tool for identifying patients at risk of LS. However, isolated cases with retained MMR protein expression despite pathogenic variants have been reported. For this reason, we support offering of germline testing to every patient with ACC, which should include at least the LS genes, *TP53, APC* and *MEN1*, as recommended by the American Society of Clinical Oncology.^77^ Identifying LS is essential not only for guiding patient management but also for enabling targeted cancer surveillance in at-risk relatives.
