# Supplementary material for: Mismatch repair deficiency and microsatellite instability in adrenocortical carcinoma
Source: ESMO Open. 2026 Feb 5;11(2):106030. doi: 10.1016/j.esmoop.2025.106030 (PMC12906187; doi:10.1016/j.esmoop.2025.106030)
Supplement: Supplementary Data [file mmc1.docx]

**Appendix to “Mismatch Repair Deficiency and Microsatellite Instability in Adrenocortical Carcinoma”.**

**DNA sequencing**

WGS as part of MASTER was carried out as described (1, 2) and germline and somatic variants in the MMR genes *MSH6*, *MSH2*, *MLH1*, and *PMS2* annotated as described below. For targeted panel analysis, data of tumour and leukocyte DNA previously sequenced with the GeneRead DNAseq Human Comprehensive Cancer Panel V2 were reanalysed (3). Targeted NGS was performed on a NextSeq500 using NextSeq Mid Output Reagent Kit V2 and 150-bp paired end reads (Illumina, San Diego, CA). For genome sequencing and targeted panel, variants in *MSH6*, *MSH2*, *MLH1*, and *PMS2* were filtered according to the following criteria: exon distance < 21 bp, MAF < 0.02, coverage at certain position > 50, variant allele frequency > 0.2, worse than synonymous, and variant balance > 0.2. The variants were classified with the use of database information (ClinVar) (4). If not already reported in databases, variants were classified with the help of Polymorphism¬Phenotyping v2 algorithm tool (PolyPhen-2) (5), and MutationTaster (6). The impact on splicing of intronic variants and variants lying in between the essential splice sides were evaluated with splice prediction tools SpliceSiteFinder-like (based on (7)), MaxEntScan (8), NNSPLICE (9), GeneSplicer (10), and Human Splicing Finder (11).

For WES, tumour and leukocyte DNA was enriched with the Twist Human Core Exome Plus Kit (Twist Bioscience, San Francisco, CA, USA). Paired-end sequencing with a length of 100 bps was performed on a NovaSeq 6000 (Illumina, San Diego, California, USA) according to the manufacturer’s protocol. Bcl2fastq, v2.20 from Illumina (San Diego, California, USA) (<https://support.illumina.com/sequencing/sequencing_software/bcl2fastq-conversion-software.html>) was used to demultiplex the reads. Adapter trimming was performed with Skewer, v0.2.2 (12). After an initial quality assessment using FastQC, v0.11.9 (http://www.bioinformatics.babraham.ac.uk/projects/fastqc), low-quality reads were trimmed with TrimGalore, v0.6.4 (http://www.bioinformatics.babraham.ac.uk/projects/trim_galore/) powered by Cutadapt, v2.8 (https://journal.embnet.org/index.php/embnetjournal/article/view/200/479). The trimmed reads were mapped to the UCSC human genome (hg19) with BWA mem, v0.7.17 (13) and sorted and indexed using Picard, v1.125 (http://broadinstitute.github.io/picard/) and SAMtools, v1.10 (14), respectively. Duplicates were marked with Picard. GATK3, v3.8, and GATK4, v4.1.9.0 (15) were used for coverage calculations and for base quality score recalibration, respectively. GATK3, GATK4 as well as Strelka, v2.9.2 (16) were used for germline variant calling. In addition, small germline indels were called using Scalpel v0.5.4 (17). MuTect2 (that is integrated in the GATK4 package), VarScan2, v2.4.4 (18), Scalpel and Strelka were used for somatic variant calling.

Somatic variants were filtered with the following criteria: variant not detectable in matched blood sample, exon distance < 21 bp, minor allele frequency (MAF) < 0.02, coverage at certain position > 100, variant allele frequency > 0.1, type of variant worse than synonymous, and variant balance > 0.2. All variants found by exome sequencing were annotated with ANNOVAR, v2019-10-24 (19) and considered if they were below a frequency of 2% in the databases 1000g2015aug_all, ExAC_nontcga_ALL, gnomAD_exome_ALL and gnomAD_genome_ALL and if the position is covered by at least 20 reads, and the alternative allele is covered by at least 8 reads and comprised at least 5% of the total reads. Each variant was visually verified using IGV, v2.12.2 (20).

**Sanger sequencing**

Pathogenic and likely pathogenic (P/LP) germline and somatic variants found in the MMR genes were further validated by Sanger sequencing. Variants of uncertain significance (VUS) of *MSH2* and *MSH6* in patients with dMMR were also validated.

DNA was amplified using the mutation-spanning primers as provided in Supplemental Table 2. Polymerase Chain Reaction (PCR) was performed on 50 ng of genomic DNA in a final volume of 20 μl using my-Budget 5x PCR-Mastermix (Bio-Budget, Krefeld, Germany). 30 cycles (denaturing at 94 °C (20 sec), annealing at 58 °C (30 sec), and elongation at 72 °C (30 sec) were passed. Direct sequencing of PCR products was performed using the QuickStart Cycle Sequencing Kit (#608120, AB Sciex, Beckman Coulter, Krefeld, Germany) on a CEQ8000 DNA Analyzer (AB Sciex), as previously described (21).

Duplication of the MSH6 gene was validated with multiplex ligation-dependent probe amplification (MLPA) method (see corresponding paragraph).

**Supplemental Figures**

**Supplemental Figure S1. A) Progression free survival (PFS) and B) overall survival (OS) in patients with and without Lynch Syndrome.**

**
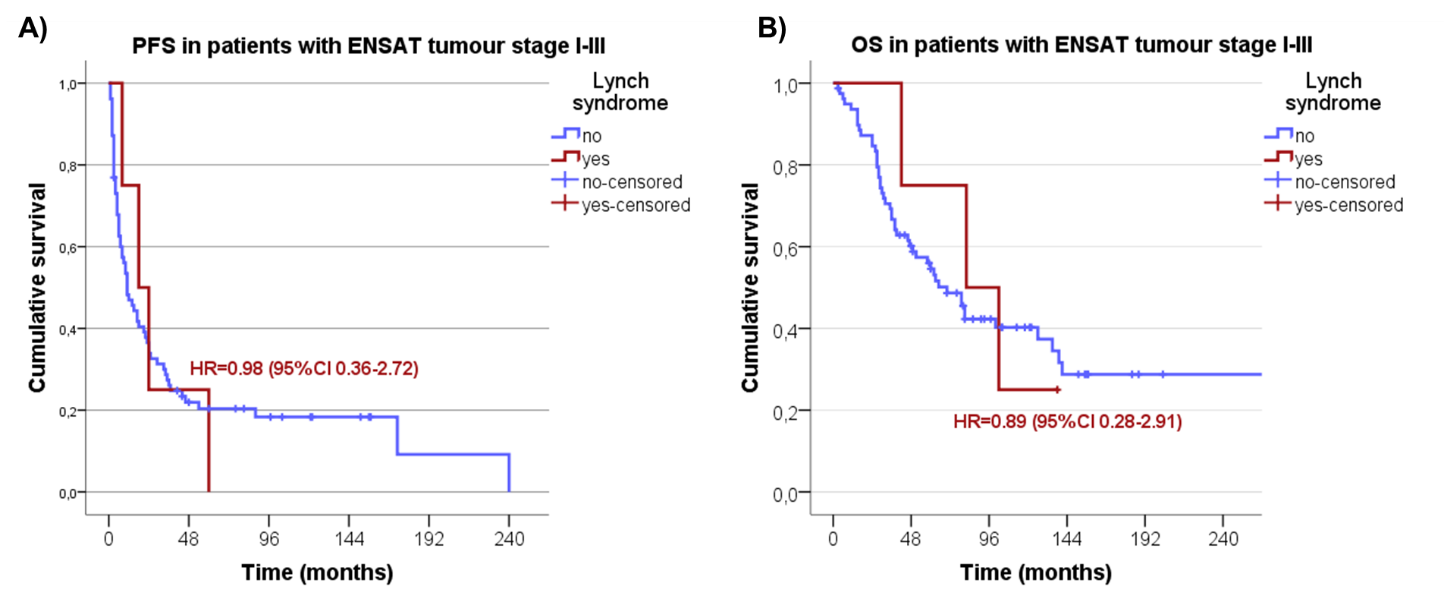
**

**Supplemental Figure S2. Time to progression (TTP) under immunotherapy.**

**
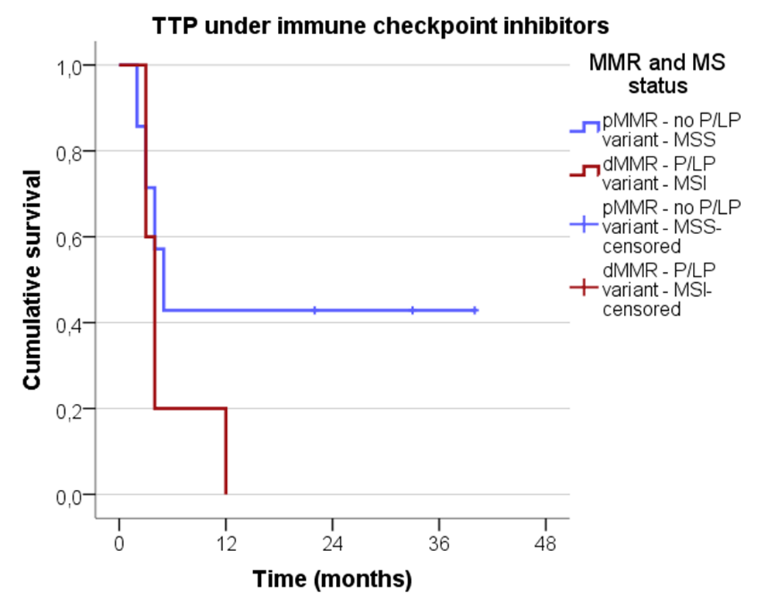
**

Abbreviation: dMMR, MMR deficiency; MSI, microsatellite instability; MSS, microsatellite stability; P/LP, pathogenic/likely pathogenic variant; pMMR, MMR proficient.

**Supplemental Table S1. The REMARK checklist.**

| **Item to be reported** | | **Reported on page n. / Table n. / Figure n.** | **Reported on section** |
| --- | --- | --- | --- |
| **INTRODUCTION** | |  |  |
| 1 | State the marker examined, the study objectives, and any pre-specified hypotheses. | p 5 | Introduction |
| **MATERIALS AND METHODS** | |  |  |
| *Patients* | |  |  |
| 2 | Describe the characteristics (e.g., disease stage or co-morbidities) of the study patients, including their source and inclusion and exclusion criteria. | p 6, Figure 1, Table 1 | Patients and methods  (patients) |
| 3 | Describe treatments received and how chosen (e.g., randomized or rule-based). | Not applicable |  |
| *Specimen characteristics* | |  |  |
| 4 | Describe type of biological material used (including control samples) and methods of preservation and storage. | p 4-6 | Patients and methods |
| *Assay methods* | |  |  |
| 5 | Specify the assay method used and provide (or reference) a detailed protocol, including specific reagents or kits used, quality control procedures, reproducibility assessments, quantitation methods, and scoring and reporting protocols. Specify whether and how assays were performed blinded to the study endpoint. | p 6-8, Appendix | Patients and methods  (immunohistochemistry; Sample collection, DNA isolation and sequencing; MMR methylation status and deletion; Microsatellite instability) and Appendix |
| *Study design* | |  |  |
| 6 | State the method of case selection, including whether prospective or retrospective and whether stratification or matching (e.g., by stage of disease or age) was used. Specify the time period from which cases were taken, the end of the follow-up period, and the median follow-up time. | p 6 | Patients and methods  (patients; statistical analysis) |
| 7 | Precisely define all clinical endpoints examined. | p 6, 8-9 | Patients and methods  (patients) |
| 8 | List all candidate variables initially examined or considered for inclusion in models. | p 6, 8-9 | Patients and methods  (patients; statistical analysis) |
| 9 | Give rationale for sample size; if the study was designed to detect a specified effect size, give the target power and effect size. | Not applicable |  |
| *Statistical analysis methods* | |  |  |
| 10 | Specify all statistical methods, including details of any variable selection procedures and other model-building issues, how model assumptions were verified, and how missing data were handled. | p 8-9 | Patients and methods  (statistical analysis) |
| 11 | Clarify how marker values were handled in the analyses; if relevant, describe methods used for cutpoint determination. | p 8-9 | Patients and methods  (statistical analysis) |
| **RESULTS** | |  |  |
| *Data* | |  |  |
| 12 | Describe the flow of patients through the study, including the number of patients included in each stage of the analysis (a diagram may be helpful) and reasons for dropout. Specifically, both overall and for each subgroup extensively examined report the numbers of patients and the number of events. | p 9-11, Figure 1 | Results |
| 13 | Report distributions of basic demographic characteristics (at least age and sex), standard (disease-specific) prognostic variables, and tumor marker, including numbers of missing values. | p 9-11, Figure 2, Table 1 | Results |
| *Analysis and presentation* | |  |  |
| 14 | Show the relation of the marker to standard prognostic variables. | Table 2, Figure 3 |  |
| 15 | Present univariable analyses showing the relation between the marker and outcome, with the estimated effect (e.g., hazard ratio and survival probability). Preferably provide similar analyses for all other variables being analyzed. For the effect of a tumor marker on a time-to-event outcome, a Kaplan-Meier plot is recommended. | Table S5, Table S6 |  |
| 16 | For key multivariable analyses, report estimated effects (e.g., hazard ratio) with confidence intervals for the marker and, at least for the final model, all other variables in the model. | Table S5, Table S6 |  |
| 17 | Among reported results, provide estimated effects with confidence intervals from an analysis in which the marker and standard prognostic variables are included, regardless of their statistical significance. | Table S5, Table S6 |  |
| 18 | If done, report results of further investigations, such as checking assumptions, sensitivity analyses, and internal validation. | p 11 | Results |
| **DISCUSSION** | |  |  |
| 19 | Interpret the results in the context of the pre-specified hypotheses and other relevant studies; include a discussion of limitations of the study. | p 12-14 | Discussion |
| 20 | Discuss implications for future research and clinical value. | p 14 | Discussion |

From: McShane LM, Altman DG, Sauerbrei W, Taube SE, Gion M, Clark GM: Reporting recommendations for tumor marker prognostic studies (REMARK). Br J Cancer. 2005 Aug 2;93(4):387–391 (22).

**Supplemental Table S2. Primers used for the Sanger sequencing**

| Primer | Patient study ID | Sequence | Evaluated cDNA change |
| --- | --- | --- | --- |
| *MLH1*-F  *MLH1*-R | 2 | TGGCTCCATTCCAAACTCCT  AAGAAGAACACATCCCACAGT | c.2179_2182del |
| *MLH1*-F  *MLH1*-R | 3 | TGGGAAGGAACCTTGTGTTT  AACCAAACTTTGCCATGAGG | c.790+4A>G |
| *MLH1*-F  *MLH1*-R | 4 | ACAGGGGTTCATTCACAGCT TGCAGTCATTTCCTTTCGGG | c.1420del |
| *MLH1*-F  *MLH1*-R | 17 | GTTTCTCACCTGCCATTCTGA ACCATCTTCCTCTGTCCAGC | c.1750G>C |
| *MSH2*-F  *MSH2*-R | 1 | CCCATTATTTATAGGATTTTGTCACTTTGT  CTTACCAAGATCTCTGGCTGC | c.1408del |
| *MSH2*-F  *MSH2*-R | 7 | GTGGCATATCCTTCCCAATGTATTG  CAAGGGTAGTAAGTTTCCCATTAC | c.2251G>A |
| *MSH2*-F  *MSH2*-R | 9 | TTCACTAATGAGCTTGCCATTC GGTATAATCATGTGGGTAACTGCAGG | c.1968C>G |
| *MSH2*-F  *MSH2*-R | 10 | TCTTTTCTGGTTAGATAATAATTGTGAG GCATTTAGGGAATTAATAAAGGGTTA | c.1552_1553delCA |
| *MSH2*-F  *MSH2*-R | 18 | TCGTTTTCCACTTGTATTTCCA GCCAGGTGACATTCAGAACA | c.1748A>G |
| *MSH2*-F  *MSH2*-R | 18 | GTGGCATATCCTTCCCAATGTATTG  CAAGGGTAGTAAGTTTCCCATTAC | c.2224G>A |
| *MSH6*-F  *MSH6*-R | 16 | AGTCGCCCTACTGTTTGGTA  GCCCAGTTGCCTTTCATGAA | c.1238G>A |
| *MSH6*-F  *MSH6*-R | 14 | CCCCAAACGATGAAGCCTC  ACTGTGTTTGGAAAATGATCACCT | c.3397A>G and  c.3245C>T |

Abbreviation: F, forward; R, reverse.

**Supplemental Table S3. Primer sequences for the evaluation of microsatellite-instability**

| Primer | Sequence | Size [bp] |
| --- | --- | --- |
| *NR-21*_F  *NR-21*_R | AAAATATTCCTACTCCGCATTCAC  GTATGTCTCCCCTGGCCTTT | 104 |
| *NR-27*_F  *NR-27*_R | CAGTTGAAAAGCCCAACGTC  GCAATGACCAATAAGCAAGTCA | 112 |
| *BAT40*_F  *BAT40*_R | CTACACCACAACCCTGCTTTT  AGTGAGCCAAGATCACACCTC | 153 |
| *KCNJ5*_F  *KCNJ5*_R | TTCCAGTCGCGTTACTACCC  CCTTGGGTCACTCCCTTTCT | 190 |

Abbreviation: F, forward; R, reverse.

**Supplemental Table S4. Details on germline and somatic pathogenic/likely pathogenic variants in MMR genes in the evaluated cohort.**

| **Germline pathogenic/likely pathogenic (P/LP) variants** | | | | | | | | | | | |
| --- | --- | --- | --- | --- | --- | --- | --- | --- | --- | --- | --- |
| **Patient ID** | **MMR protein by IHC** | **Protein deficiency** | **Microsatellite status by plex PCR** | **Sequencing method** | **NCBI gene ID (HGNC)** | **HGVS** | **Functional effect** | **Classification** | **Personal history of other tumours** | **Control VAF** | **Tumor VAF** |
| 3 | dMMR | MLH1, PMS2 | MSS | WGS | 4292 | NM_000249.3(MLH1): c.790+4A>G p.(?) | Splice | P/LP | Unaffected | 0,5 | 0,95 |
| 9 | dMMR | MSH2 | MSI | targeted NGS | 4436 | NM_000251.2(MSH2): c.1968C>G p.Tyr656* | Stop gain | P/LP | Unaffected | 0,48 | 0,77 |
| 10 | dMMR | MSH2, MSH6 | MSI | WGS | 4436 | NM_000251.3(MSH2): c.1552_1553delCA p.Gln518Valfs*10 | Frameshift | P/LP | Colon Cancer | 0,47 | 0,85 |
| 13 | dMMR | MSH6 | MSS | WGS | 2956 | Chr2(MSH6):g.48029043_48032607dup | CNV - duplication (exons 5-6) | P/LP | Breast cancer | het | hom |
| 14 | dMMR | MSH6 | MSS | targeted NGS | 2956 | NM_000179.2(MSH6): c.3245C>T p.Pro1082Leu | Missense | VUS | Unaffected | 0,36 | 0,74 |
| 16 | pMMR | - | MSS | WGS | 2956 | NM_000179.3(MSH6): c.1238G>A p.Trp413* | Stop gain | P/LP | Unaffected | 0,48 | 0,6 |
| 18 | pMMR | - | MSI | WGS | 4436 | NM_000251.3(MSH2): c.1748A>G p.Asn583Ser | Missense | VUS | Pheochromocytoma | 0,59 | 0,33 |
|  |  |  |  |  | 4436 | NM_000251.3(MSH2): c.2224G>A p.Asp742Asn | Missense | VUS |  | 0,49 | 0,87 |
| **Somatic oncogenic/likely oncogenic (O/LO) variants** | | | | | | | | | | | |
| **Patient ID** | **MMR protein by IHC** | **Protein deficiency** | **Microsatellite status by plex PCR** | **Sequencing method** | **NCBI gene ID (HGNC)** | **HGVS** | **Functional effect** | **Classification** | **Personal history of other tumours** | **Control VAF** | **Tumor VAF** |
| 1 | dMMR | MLH1, PMS2, MSH2, MSH6 | MSS | targeted NGS | 4436 | NM_000251.2(MSH2): c.1408del p.Val470* | Nonsense | P/LP | Unaffected | 0 | 0,15 |
| 2 | dMMR | MLH1, PMS2, MSH6 | MSI | targeted NGS | 4291 | NM_000249.3(MLH1): c.2179_2182del p.His727Phefs*55 | Frameshift | P/LP | Unaffected | 0 | 0,21 |
| 4 | dMMR | MLH1, PMS2 | MSS | targeted NGS | 4291 | NM_000249.3(MLH1): c.1420del p.Arg474Glyfs*17 | Frameshift | P/LP | Unaffected | 0 | 0,58 |
| 7 | dMMR | PMS2 | MSS | targeted NGS | 4436 | NM_000251(MSH2): c.2251G>A p.Gly751Arg | Missense | P/LP | Unaffected | 0 | 0,82 |
| 14 | dMMR | MSH6 | MSS | targeted NGS | 2956 | NM_001281492.2(MSH6): c.3007A>G p.Thr1003Ala | Missense | P/LP | Unaffected | 0 | 0,41 |
| 17 | pMMR | - | MSS | WGS | 4291 | NM_000249.4(MLH1): c.G1750C>G p.Asp584His | Missense | P/LP | Unaffected | 0,18 | 0 |

**Supplemental Table S5. Univariate and multivariate Cox regression analysis for progression-free survival (PFS) in patients with ENSAT tumour stage I-III.**

|  |  | Median PFS | Univariate analysis | | | Multivariate analysis | | |
| --- | --- | --- | --- | --- | --- | --- | --- | --- |
| Variables | **n** | **months** | **HR** | **95% CI** | ***p*** | **HR** | **95% CI** | ***p*** |
| MMR status:   - pMMR - dMMR | 69  13 | 14  8 | 1  1.21 | 0.63-2.32 | 0.56 | 1  1.44 | 0.45-4.56 | 0.54 |
| Sex:   - M - F | 35  47 | 15  11 | 1  1.05 | 0.65-1.71 | 0.83 | - |  |  |
| Age:   - <50 yrs - ≥50 yrs | 44  38 | 11  12 | 1  1.21 | 0.74-1.96 | 0.45 | - |  |  |
| Hormone excess:   - Inactive - GC ± others - Other steroids | 18  31  9 | 34  8  35 | 1  2.71  1.83 | 1.30-5.65  0.72-4-64 | 0.008  0.20 | 1  2.54  1.66 | 1.02-6.33  0.57-4.84 | 0.04  0.35 |
| ENSAT tumour stage:   - I-II - III | 54  28 | 22  6 | 1  1.69 | 1.02-2.80 | 0.04 | 1  1.06 | 0.50-2.26 | 0.87 |
| Resection status:   - R0-RX - R1-R2 | 71  9 | 14  3 | 1  2.62 | 1.28-5.35 | 0.008 | 1  2.14 | 0.63-7.20 | 0.22 |
| ki67:   - <20% - ≥20% | 46  30 | 24  5 | 1  3.57 | 2.08-6.16 | <0.001 | 1  3.14 | 1.44-6.83 | 0.004 |
| S-GRAS score:   - 0-3 - 4-9 | 51  23 | 23  4 | 1  3.88 | 2.18-6-69 | <0.001 | 1  2.05 | 0.79-5.29 | 0.14 |

Abbreviation: CG, glucocorticoids; dMMR, MMR deficiency; HR, hazard ratio; n, number of patients; pMMR, MMR proficient; yrs, years; 95%CI, 95 % confident interval.

**Supplemental Table S6. Univariate and multivariate Cox regression analysis for overall survival (OS) in patients with ENSAT tumour stage I-III.**

|  |  | Median OS | Univariate analysis | | | Multivariate analysis | | |
| --- | --- | --- | --- | --- | --- | --- | --- | --- |
| Variables | **n** | **months** | **HR** | **95% CI** | ***p*** | **HR** | **95% CI** | ***p*** |
| MMR status:   - pMMR - dMMR | 70  13 | 70  82 | 1  1.15 | 0.54-2.46 | 0.71 | 1  2.22 | 0.57-8.69 | 0.25 |
| Sex:   - M - F | 35  48 | 102  62 | 1  1.32 | 0.75-2.31 | 0.34 | - |  |  |
| Age:   - <50 yrs - ≥50 yrs | 44  39 | 58  81 | 1  0.23 | 0.41-1.25 | 0.23 | - |  |  |
| Hormone excess:   - Inactive - GC ± others - Other steroids | 18  31  9 | not reached  38  139 | 1  6.91  3.37 | 2.37-20.11  0.95-11.99 | <0.001  0.06 | 1  7.81  4.18 | 2.37-25.74  1.06-16.54 | 0.001  0.04 |
| ENSAT tumour stage:   - I-II - III | 54  29 | 100  36 | 1  1.99 | 1.14-3.45 | 0.01 | 1  1.15 | 0.49-2.71 | 0.75 |
| Resection status:   - 0-X - 1-2 | 72  9 | 79  36 | 1  2.41 | 1.12-5.21 | 0.02 | 1  1.53 | 0.43-5.41 | 0.50 |
| ki67:   - <20% - ≥20% | 47  30 | 126  39 | 1  2.60 | 1.46-6.62 | 0.001 | 1  2.19 | 0.95-5.05 | 0.06 |
| S-GRAS score:   - 0-3 - 4-9 | 52  23 | 82  31 | 1  2.59 | 1.42-4.72 | 0.002 | 1  2.21 | 0.74-6.60 | 0.15 |

Abbreviation: CG, glucocorticoids; dMMR, MMR deficiency; HR, hazard ratio; n, number of patients; pMMR, MMR proficient; yrs, years; 95%CI, 95 % confident interval.

**Supplemental Table S7. Summary of the literature in adult patients with adrenocortical carcinoma and Lynch syndrome or evaluation of the MMR / MSI system.**

| **Cohort studies** | | | | | | | | |
| --- | --- | --- | --- | --- | --- | --- | --- | --- |
| **Reference** | **Study** | **n of patients with LS in cohort of ACC** | **n of ACC in patients with LS** | **P/LP germline variants in MMR genes** | **Hypermethylation of MLH1** | **O/LO somatic variants in MMR genes** | **MMR protein expression at IHC** | **MSI status** |
| Current study | Retrospective in patients with sporadic ACC | 5 / 109 (4.6%) | - | *MLH1* (n=1)  *MSH2* (n=2)  *MSH6* (n=2) | n=1 / 15 with dMMR | *MLH1* (n=3)  *MSH2* (n=2)  *MSH6* (n=1) | dMMR (n=15)  pMMR (n=94, including 3 with *MMRs* variants /MSI) | MSI (n=4 / 99, including 2 pts with germline *MMR* variants).  MSS (n=95 / 99) |
| Scatolini et al. 2024 [43] | Retrospective in patients with sporadic ACC | 1 / 150  (0.6%) | - | *MSH2* (n=1)  (only *MSH2* and *MSH6* were tested) | - | - | dMMR (MSH6 loss) | - |
| Domenech et al. 2021 [28] | Cohort in patients with LS | - | 3 / 643 (0.5%) | All *MSH2* | - | - | All dMMR  (MSH2 + MSH6 loss) | MSI (n=1)  MSS (n=2) |
| Pozdeyev et al. 2021 [59] | Retrospective in 364 patients with ACC | - | - | - | - | In 29 / 364 pts:  *MLH1* (n=3)  *MSH2* (n=14)  *MSH6* (n=14)  PMS2 (n=1) | - | MSI (n=1 / 272, in pt without *MMR* variants)  MSS (n=267 / 272)  MSI ambiguous (n=4 / 272, including 3 pts with *MMR* variants) |
| Raymond et al. 2013 [27] | Consecutive series of ACC (cohort 1) + registry of patients with LS (cohort 2) | 3 / 94 (3.2%) | 2 / 135 (1.5%) | *MLH1* (n=1)  *MSH2* (n=3)  *MSH6* (n=1) | - | - | dMMR (n=3 / 4, including 2 pts with MSH2 + MSH6 loss and 1 pt with MLH1 + PMS2)  pMMR (n=1 / 4) | MSS (n=4 / 4) |
| **Case reports / case series** | | | | | | | | |
| **Reference** | **Study** | **n of patients with LS in cohort of ACC** | **n of ACC in patients with LS** | **P/LP germline variants in MMR gene** | **Hypermethylation of MLH1** | **O/LO somatic variants in MMR gene** | **MMR protein expression at IHC** | **MSI status** |
| Kalomeris et al. 2024 [44] | Case series | 1 / 5 | - | *MSH6* | - | - | - | MSI score low |
| Ahuja et al. 2024 [45] | Case report | 1 | - | *MSH2* | - | negative | - | - |
| Raygada et al. 2021 [46] | Case report | - | 1 | *MSH2* (associated with *RET* variant) | - | - | - | Inconclusive |
| Shetty et al. 2020 [47] | Case report | - | 1 | *MSH2* | - | - | dMMR  (MSH2 + MSH6 loss) | - |
| Kaur et al. 2019 [48] | Case report | - | 1 | *MSH6* | - | - | - | - |
| Casey et al. 2018 [49] | Case report | - | 1 | *MSH2* | - | - | dMMR  (MSH2 + MSH6 loss) | - |
| Wright et al. 2018 [50] | Case report | - | 1 | *MSH2* | - | - | dMMR  (MSH2 loss + MSH6 patchy loss) | - |
| Challis et al 2016 [51] | Case series in a family with LS | - | 2 | *MSH2* | - | - | Both dMMR  (MSH2 + MSH6 loss) | - |
| Karamurzin et al 2012 [52] | Case series on unusual tumour in LS | - | 1 | *MSH2* | - | - | dMMR  (MSH2 + MSH6 loss) | MSS |
| Medina-Arana et al. 2011 [53] | Case report | - | 1 | *MSH2* | - | - | dMMR (MSH2 loss) | MSS |
| Broaddus et al. 2004 [54] | Case series on unusual tumour in LS | - | 1 | *MSH2* | - | - | dMMR (MSH2 loss) | MSS |
| Berends et al. 2000 [55] | Case report | - | 1 | *MSH2* | - | - | pMMR | MSS |

Cohort and case series studies on LS and ACC as well as general genetic studies on ACC evaluating the MMR/MSI system were included in this table. Only pathogenic and likely pathogenic germline and somatic variants were considered. Abbreviation: ACC, adrenocortical carcinoma; dMMR, MMR deficiency; IHC, immunohistochemistry; LS, Lynch syndrome; MMR, DNA mismatch repair; MSI, microsatellite instability; MSS, microsatellite stable; O/OL, oncogenic/likely oncogenic; PCR, Polymerase chain reaction; pMMR, MMR proficient (no loss); P/LP, pathogenic/likely pathogenic; -, not available/not applicable/analysis not performed.

**References to Supplementary**

1. Horak P, Heining C, Kreutzfeldt S, Hutter B, Mock A, Hullein J, et al. Comprehensive Genomic and Transcriptomic Analysis for Guiding Therapeutic Decisions in Patients with Rare Cancers. Cancer Discov. 2021;11(11):2780-95.

2. Jahn A, Rump A, Widmann TJ, Heining C, Horak P, Hutter B, et al. Comprehensive cancer predisposition testing within the prospective MASTER trial identifies hereditary cancer patients and supports treatment decisions for rare cancers. Ann Oncol. 2022;33(11):1186-99.

3. Lippert J, Appenzeller S, Liang R, Sbiera S, Kircher S, Altieri B, et al. Targeted Molecular Analysis in Adrenocortical Carcinomas: A Strategy Toward Improved Personalized Prognostication. J Clin Endocrinol Metab. 2018;103(12):4511-23.

4. Landrum MJ, Lee JM, Benson M, Brown GR, Chao C, Chitipiralla S, et al. ClinVar: improving access to variant interpretations and supporting evidence. Nucleic Acids Res. 2018;46(D1):D1062-D7.

5. Adzhubei IA, Schmidt S, Peshkin L, Ramensky VE, Gerasimova A, Bork P, et al. A method and server for predicting damaging missense mutations. Nat Methods. 2010;7(4):248-9.

6. Schwarz JM, Cooper DN, Schuelke M, Seelow D. MutationTaster2: mutation prediction for the deep-sequencing age. Nat Methods. 2014;11(4):361-2.

7. Shapiro MB, Senapathy P. RNA splice junctions of different classes of eukaryotes: sequence statistics and functional implications in gene expression. Nucleic Acids Res. 1987;15(17):7155-74.

8. Yeo G, Burge CB. Maximum entropy modeling of short sequence motifs with applications to RNA splicing signals. J Comput Biol. 2004;11(2-3):377-94.

9. Reese MG, Eeckman FH, Kulp D, Haussler D. Improved splice site detection in Genie. J Comput Biol. 1997;4(3):311-23.

10. Pertea M, Lin X, Salzberg SL. GeneSplicer: a new computational method for splice site prediction. Nucleic Acids Res. 2001;29(5):1185-90.

11. Desmet FO, Hamroun D, Lalande M, Collod-Beroud G, Claustres M, Beroud C. Human Splicing Finder: an online bioinformatics tool to predict splicing signals. Nucleic Acids Res. 2009;37(9):e67.

12. Jiang H, Lei R, Ding SW, Zhu S. Skewer: a fast and accurate adapter trimmer for next-generation sequencing paired-end reads. BMC Bioinformatics. 2014;15:182.

13. Li H, Durbin R. Fast and accurate short read alignment with Burrows-Wheeler transform. Bioinformatics. 2009;25(14):1754-60.

14. Li H, Handsaker B, Wysoker A, Fennell T, Ruan J, Homer N, et al. The Sequence Alignment/Map format and SAMtools. Bioinformatics. 2009;25(16):2078-9.

15. McKenna A, Hanna M, Banks E, Sivachenko A, Cibulskis K, Kernytsky A, et al. The Genome Analysis Toolkit: a MapReduce framework for analyzing next-generation DNA sequencing data. Genome Res. 2010;20(9):1297-303.

16. Kim S, Scheffler K, Halpern AL, Bekritsky MA, Noh E, Kallberg M, et al. Strelka2: fast and accurate calling of germline and somatic variants. Nat Methods. 2018;15(8):591-4.

17. Fang H, Wu Y, Narzisi G, O'Rawe JA, Barron LT, Rosenbaum J, et al. Reducing INDEL calling errors in whole genome and exome sequencing data. Genome Med. 2014;6(10):89.

18. Koboldt DC, Zhang Q, Larson DE, Shen D, McLellan MD, Lin L, et al. VarScan 2: somatic mutation and copy number alteration discovery in cancer by exome sequencing. Genome Res. 2012;22(3):568-76.

19. Wang K, Li M, Hakonarson H. ANNOVAR: functional annotation of genetic variants from high-throughput sequencing data. Nucleic Acids Res. 2010;38(16):e164.

20. Robinson JT, Thorvaldsdottir H, Winckler W, Guttman M, Lander ES, Getz G, et al. Integrative genomics viewer. Nat Biotechnol. 2011;29(1):24-6.

21. Altieri B, Sbiera S, Herterich S, De Francia S, Della Casa S, Calabrese A, et al. Effects of Germline CYP2W1*6 and CYP2B6*6 Single Nucleotide Polymorphisms on Mitotane Treatment in Adrenocortical Carcinoma: A Multicenter ENSAT Study. Cancers (Basel). 2020;12(2).

22. McShane LM, Altman DG, Sauerbrei W, Taube SE, Gion M, Clark GM, et al. REporting recommendations for tumour MARKer prognostic studies (REMARK). Br J Cancer. 2005;93(4):387-91.
